# Application of Artificial Intelligence In Drug-target Interactions Prediction: A Review

**DOI:** 10.1038/s44385-024-00003-9

**Published:** 2025-01-13

**Authors:** Qian Liao, Yu Zhang, Ying Chu, Yi Ding, Zhen Liu, Xianyi Zhao, Yizheng Wang, Jie Wan, Yijie Ding, Prayag Tiwari, Quan Zou, Ke Han

**Affiliations:** 1 School of Computer and Information Engineering, Harbin University of Commerce, Harbin, China; 2 Yangtze Delta Region Institute (Quzhou), University of Electronic Science and Technology of China, Quzhou, China; 3 Institute of Fundamental and Frontier Sciences, University of Electronic Science and Technology of China, Chengdu, China; 4 the Laboratory for Space Environment and Physical Sciences, Harbin Institute of Technology, Harbin, China; 5 School of Information Technology, Halmstad University, Halmstad, Sweden; 6 Pharmaceutical Engineering Technology Research Center, Harbin University of Commerce, Harbin, China

**Keywords:** Computational biology and bioinformatics, Drug discovery

## Abstract

Predicting drug-target interactions (DTI) is a complex task. With the introduction of artificial intelligence (AI) methods such as machine learning and deep learning, AI-based DTI prediction can significantly enhance speed, reduce costs, and screen potential drug design options before conducting actual experiments. However, the application of AI methods also faces several challenges that need to be addressed. This article reviews various AI-based approaches and suggests possible future directions.

## Introduction

Drug research and development have traditionally incurred considerable input costs in terms of time and capital. Furthermore, predicting experimental results has proven to be a challenge. However, the introduction of artificial intelligence (AI) methods in recent years has offered an opportunity to mitigate these issues. By leveraging AI for computational analysis and prediction, the wet experimental method can be considerably improved, leading to expedited drug research and development processes and the avoidance of unnecessary expenses^1^.

Drug-target interaction (DTI) identification and prediction is of great significance in drug discovery and practical applications in biomedical fields, mainly including drug repositioning, where prediction of drug-protein interactions can help to discover new applications of existing drugs, enable reassessment of marketed drugs, search for new targets related to other diseases, and accelerate the exploration and development of new indications; and new drug discovery^2^. By predicting the interactions between drugs and specific proteins, it can provide guidance for the design and discovery of new drugs. This is important for discovering potential drug candidate compounds, accelerating the drug development process, and reducing costs and risks; Side effect prediction, where interactions between drugs and proteins may lead to drug side effects and adverse reactions. By predicting drug-target interactions, potential side effects can be detected in advance, thus enhancing the safety assessment and monitoring of drugs. And through these predicted results, it can help researchers better explore and understand the mechanism of action of drugs, revealing how drugs regulate biological processes to produce therapeutic effects. In turn, it may be possible to find individualized therapies based on the genetic, phenotypic and environmental characteristics of individuals to provide precise treatment solutions. In particular, in 2020, Zhou et al.^3^ modeled a network-based DTI prediction system (TargetPredict) analysis based on genes, diseases, genetic relationships, drugs, and side effects together and quickly found that the prescription of liraglutide, a drug used in type I diabetes, was significantly associated with a reduced risk of AD diagnosis. In 2022, Zhang et al.^4^ verified that the Transformer-based model performed well on large-scale infections in COVID-19 as well as drug repositioning experiments in Alzheimer’s disease (AD). In 2023, Lee et al.^5^ based on the MT-DTI approach on the prediction of potential TRPV1 antagonists looked for triclopyr as the active ingredient, helping to contribute to improving skin sensitivity in clinical practice.

Therefore, these examples illustrate the feasibility and great potential of AI-based methods^6^ for predicting drug target pairwise interactions. Artificial intelligence methods, whether they figure out how to exclude as many true negatives as possible from the predicted drug-target pairings or screen out true positives as accurately as possible, can reduce the cost of trial-and-error in subsequent wet-lab work and increase efficiency.

The data in DTI includes various types of information such as molecular structures of drugs and proteins, interaction details, clinical manifestations, drug side effects, and more. Among these, known interactions between drugs and targets are significantly sparse compared to unknown interactions. Therefore, the most common issue encountered in this field is the imbalance between positive and negative samples, making it challenging to achieve optimal model performance. This undoubtedly presents a major challenge. Simultaneously, the integration and feature extraction of textual information, along with the emergence of AlphaFold^7^, have sparked increasing interest in the study of protein folding structures. The inclusion of these three-dimensional structures raises questions about whether they can positively impact model predictions and how to maximize these potential benefits. With the advent of generative AI (GAI), there’s a new foundation for designing drug molecules from scratch, prompting consideration and experimentation on what preparations are needed to effectively generate viable drug molecules using GAI. In recent years, quantum chemistry has also garnered attention and discussion for its feasibility in optimizing complex structures at the particle level and studying enzymatic catalysis reactions. Finally, the arrival of large-scale models enables rapid dialog and communication, allowing us to swiftly obtain numerous solutions. Thus, exploring how to harness the powerful reasoning capabilities of large language models (LLM) to integrate drug discovery tasks represents a new frontier.

We used Google Scholar to broadly gather papers by searching keywords such as “drug-target interactions (DTI)”, “artificial intelligence”, “machine learning”, and “deep learning”, and then arranged them by year. We reclassified and filtered these papers based on keywords like “dataset”, “3D structure”, “multimodal”, “feature fusion”, “graph neural networks”, “Transformer”, “attention mechanism”, “contrastive learning”, “generative AI”, “knowledge graph”, and “meta-path”. Additionally, we explored the feasibility of emerging research directions, including “AlphaFold3”, “quantum chemistry”, and “large models”. Since drug performance analysis relies on clinical data, we also included a discussion on data privacy and security to address public concerns. Ultimately, we selected 100 representative references that enable in-depth discussion. These retrieved articles came from the following different journals and conferences: Nature Reviews Bioengineering, Nature Reviews Drug Discovery, Journal of Chemical Information and Modeling, Bioinformatics, Briefings in Bioinformatics, Methods, IEEE/ACM Transactions on Computational Biology and Bioinformatics, BMC Bioinformatics, IEEE International Conference on Bioinformatics and Biomedicine (BIBM), International Conference on Bioinformatics and Biomedical Technology (ICBBT), International Conference on Intelligent Computing (ICIC), and so on.

The citations in the methods section of this paper are from new and some classical methods presented in journals and conferences in the field of drug-target relevance prediction in the last five years. These tasks are mainly concerned with the prediction of drug-target interactions and drug-target affinity (DTA). The data cited are from publicly available datasets and online sources. The methods discussed include traditional machine learning and deep learning approaches, and advances in computational virtual prediction techniques such as Generative Artificial Intelligence (GAI), quantum chemistry, and Large Language Modeling (LLM) are presented. We hope that these cutting-edge approaches will expand the possibilities for drug discovery and apply recent developments in these technologies to the field of drug target relevance prediction. Through advances in fundamental methods, we aim to break through bottlenecks and contribute to the advancement of drug discovery.

## Fundamentals and relevant background of DTI prediction

### Problem analysis

The conventional drug discovery process involves step-by-step hypotheses and derivations through wet experiments followed by design and experimentation. However, this experimental process does not work all at once; the validation of hypotheses, derivation process, deviations in design, and errors in actual experimentation can lead to failures at each step. This step requires a significant amount of time and sizable experimental funding only once; if repeated multiple times, the time and money consumed may be incalculable. With the development and application of AI methods in diverse forecasting fields, these methods have also been used for DTI forecasting.

### Artificial intelligence methods for DTI prediction

**Fig. 1:**
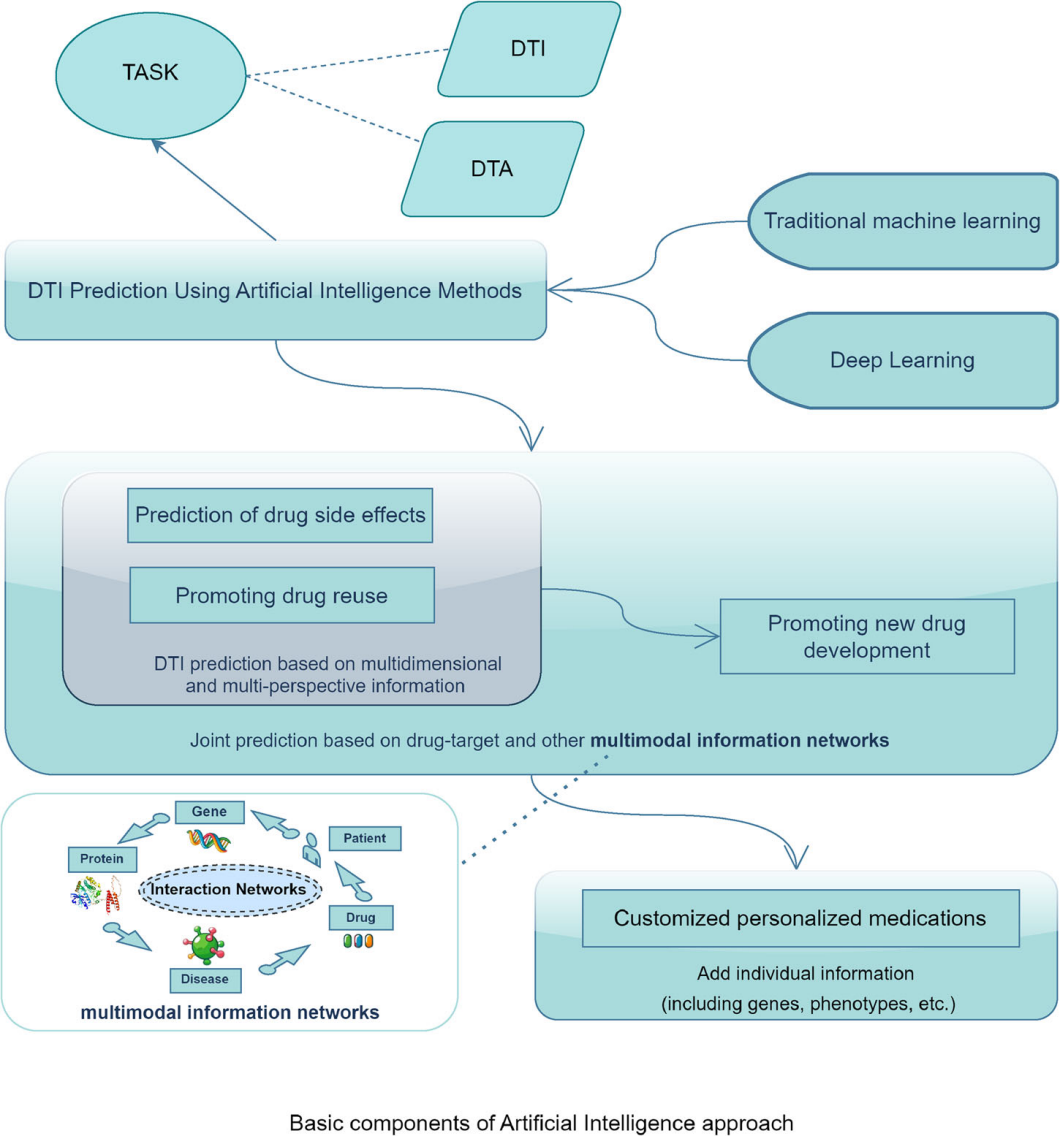
Basic components of AI-based approach. The prediction of drug–target interactions and the affinity relationships of drug–targets through machine learning and deep learning methodologies enables a multi-faceted approach that is not constrained to a single information source. This approach concurrently utilizes multiple factors, including drugs, diseases, genes, proteins, and patient characteristics, facilitating predictions that integrate not only sequence data but also images and three-dimensional models. Consequently, this methodology aims to enhance predictive accuracy while optimizing prediction speed as much as possible.

The artificial intelligence method is a computerized method used to complete the hypothesis derivation and design process of drug development work before the wet experiment, which becomes the preparation for the wet experiment and can offer reference and feedback guidance for subsequent clinical side-effect monitoring. Therefore, AI and wet method experiments can be combined for the entire drug development process. The DTI prediction process based on the AI method is illustrated in Fig. 1.

The use of AI to predict drug-target interactions has long attracted the attention of researchers in various countries as a critical technological tool to assist in drug discovery and development, and has been continuously improved with the development of AI methods. According to the order of technological advancement, from conventional docking simulation methods, statistical econometric analysis methods, machine learning methods, deep learning methods, and the future of AI4science in the context of the big model in full swing, there are diverse linkages between various industries and the big model. Compared to the traditional wet experiment-only approach to study drug-target interrelationships, the front-step AI approach is a big trend in DTI prediction before conducting the final wet experiment.

The efficacy of a drug is ascribed to its interaction with its corresponding target. Consequently, research on AI methods aims to address how to establish a correspondence between the drug and the target. Present research tackles this issue in two main approaches. Firstly, it explores the existence of a correlation between the drug and the target and treats the relationship as a “0/1” problem. This classification problem can be resolved through binary classification or by assigning a candidate ranking. Secondly, it utilizes the affinity coefficient relationship between the drug and the target to evaluate and normalize their association as a “0/1” problem. This binary classification problem can be solved by considering the relationship between the drug and target. Additionally, it is possible to also consider the relationship between drug targets as a regression issue, by normalizing the relationship to a value interval between 0 and 1. In summary, the study of drug-target interaction relationships can be divided into two types of tasks: one involves classification prediction of drug-target interactions through qualitative analysis, while the other involves quantitative analysis in the prediction of drug-target affinity.

Complex and difficult input data were encountered in both research tasks. To study drug-target interactions, key information, including drug, target, and known drug-target interrelationship data is required. Drugs are usually used for chemical coding and drug fingerprinting, and proteins contain sequence information, 3D structure information, residue information, and several other information categories. Additionally, inter- and intra-class associations can be explored using inter-drug and inter-target correlation datasets. DTIs predictions can be based on one or more of the aforementioned modal datasets.

### Data for the study

#### Data sources and data types

Diverse types of information, such as the type of sample data used in DTI, its structure, dimensionality, and the proportion of positive and negative sample components, may affect the prediction performance of the model. Therefore, it is critical to select appropriate data as sample inputs for model prediction.

In drug-target interaction prediction, commonly used data types include drugs, proteins, diseases^8^, side effects, genes, drug-target interaction information, and protein interaction information, including data on the genetic properties of a specific disease that will be involved in certain methods^3^ or observations on a certain sample^4^. The sources of these data include online platforms, biomedical literature, expert opinions, laboratory data, internal information of biomedical companies, and clinical information. Further research can be conducted by selecting the type of data processed by the model inputs from among these different multimodal data sources.

**Fig. 2:**
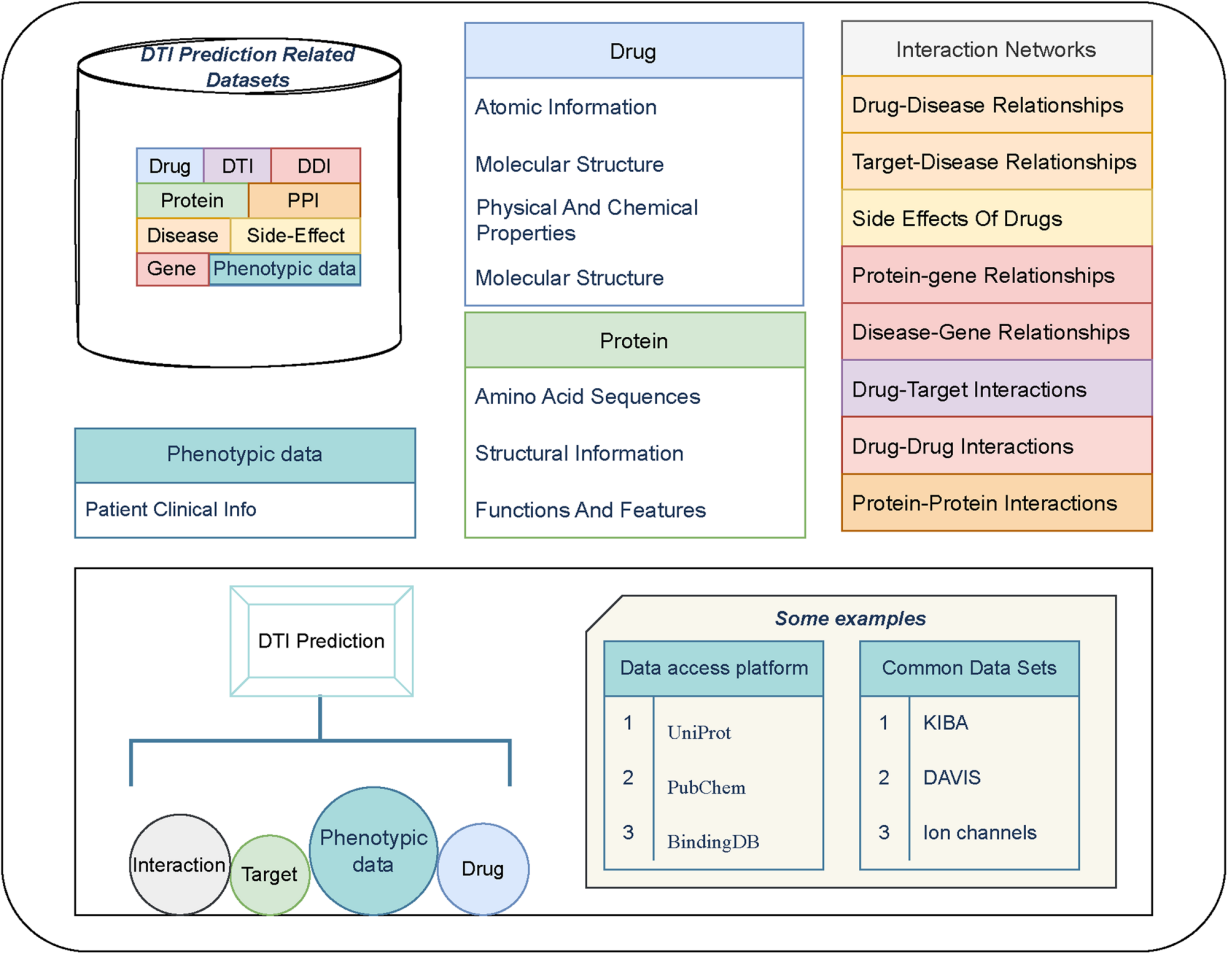
Introduction to the data resources. The prediction of relationships between drug–targets requires the use of a number of databases related to drugs and targets, which contain two main categories of data, (1) the characteristics of the drug and target data themselves, and (2) the associative information between the drug and the target, including known drug–drug interactions, drug–target interactions, drug–side effect relationships, and phenotypic data. Common data sources for these data include a number of online websites and public datasets.

Commonly used datasets include the gold standard datasets Nuclear Receptor(NR), G Protein-Coupled Receptor(GPCR), Ion Channel(IC), Enzyme(Es)^9^ and other public datasets, such as Davis^10^ and KIBA^11^, and some studies have integrated data from data platforms, including BindingDB^12^, Uniport (Uniport: https://www.uniport.org), and PubChem(PubChem: https://pubchem.ncbi.nlm.nih.gov). Drug molecule representations include Simplified Molecular Input Line Entry System(SMILES) and drug molecule maps, and protein representations include sequence, FASTA, PDB, and protein contact maps. These data can be obtained directly from data resource-providing platforms or converted and processed using tools, such as rdkit(rdkit: https://rdkit.org). Collectively, these complex data formats and multiple data sources form a large knowledge network for DTI prediction. The basic relationships between these data are presented in Fig. 2.

These data can be categorized into one-dimensional (1D), two-dimensional (2D), and three-dimensional (3D) data based on their dimensions, including descriptive text, binary images, and three-dimensional models. The specific descriptions are depicted in Table 1.

**Table 1:** Data Dimension Classification

Data dimensions	Data description
1D	Contains molecular sequence information on drugs and targets, additional information on diseases, side effects, clinical manifestations, and other input information in text form such as genetic information.
2D	Contains drug molecule maps, protein contact maps, drug-target interaction networks and other relevant interaction networks, as well as other input information in the form of images.
3D	Contains 3D models of structural information about the drug and target, as well as other forms of presented 3D input information.

In recent years, two types of data developments for prediction have drawn attention: quantum chemistry data and AlphaFold-predicted protein structures.

1. Quantum Chemistry Data(Quantum Chemistry Data: https://cadd-tutorial.readthedocs.io/zh-cn/latest/QM.html): This includes various parameters for computable molecules such as molecular structure, bonding features, total system energy, molecular information for each orbital, molecular interactions, various spectra, vibrational spectra, electromagnetic spectral properties, and reaction transition states. Quantum chemical descriptors serve as descriptors for quantitative structure-activity relationships, describing internal molecular properties at the particle level, including total energy, ionization energy, molecular heat of formation, octanol-water partition coefficient, dipole moment, molecular polarizability, molecular weight, van der Waals volume, and some electrical parameters, etc^13^. Based on this foundation, it is possible to predict the sites of halogenation reactions, nucleophilic substitution reactions, and sites where affinity occurs. If we can handle aligned quantum chemistry data for particle-level prediction tasks, we can optimize our predictive models from an interpretable perspective, thereby improving the generalization performance of the models.
2. AlphaFold^14^: Since the debut of the first generation AlphaFold in the Critical Assessment of Protein Structure Prediction competition at the beginning of 2020, which showcased its powerful protein structure prediction capabilities and garnered attention, AlphaFold has undergone two major upgrades. In 2024, AlphaFold3 was released, successfully predicting the structures and interactions of almost all biomolecules (proteins, DNA, RNA, ligands, etc.). These successful predictions of chemical structures undoubtedly enrich the quantity of structural samples and strengthen the robustness of models if applied to structure-based DTI (Drug-Target Interaction) and DTA (Drug-Target Affinity) prediction tasks.

#### Transition from single- to multi-modality

**Fig. 3:**
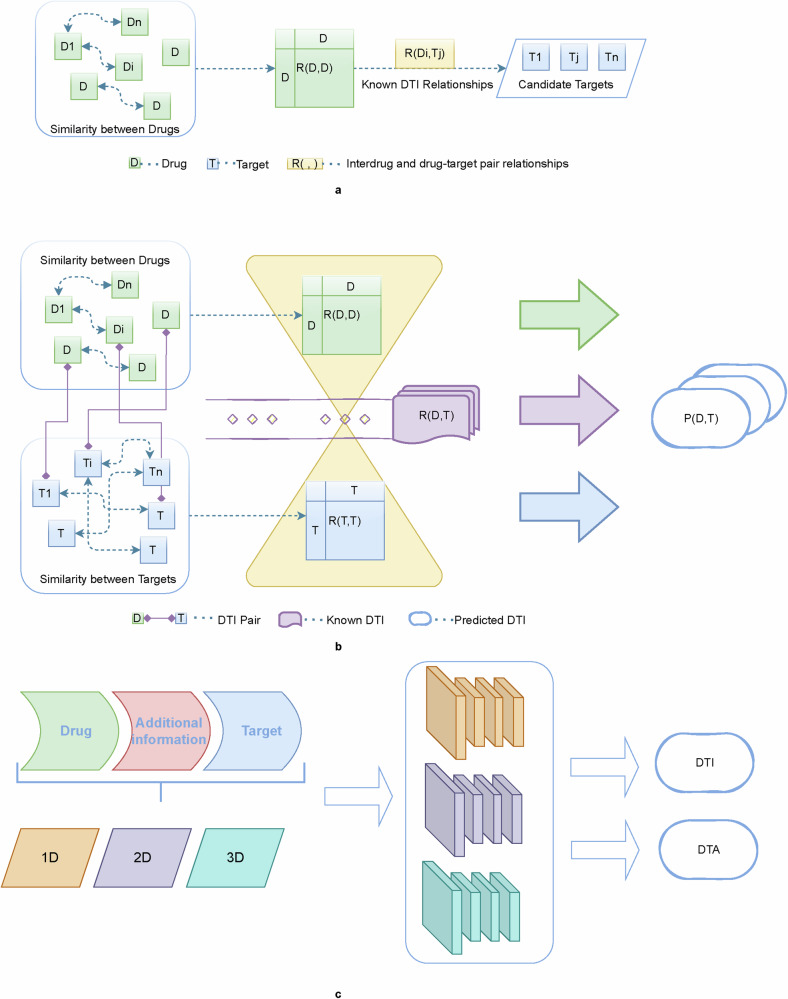
Multimodal network evolution for DTI prediction. **a** approaches that focus only on drug-side information; **b** approaches that focus only on known relationships for drug-target interactions; and **c** approaches that focus on the drug and target as well as other additional information. Models use data modalities that gradually move from single to multimodal.

Currently, with in-depth research on drug-target interactions, the conventional one-drug-one-target model is no longer applicable. One protein may correspond to multiple targets^15^, and there may also be synergies between drugs^16^. Integrating big data and complex heterogeneous networks is conducive to expanding the understanding of drug-target interactions. To better mine the relevant information flow diagrams for unimodal and multimodal are depicted in Fig. 3.

Most previous DTI prediction methods only considered the structural relationships between drug compounds, whereas target-side information was not sufficiently considered. Conversely, researchers nowadays are more focused on mining both drug and target information simultaneously to obtain as much potential correlation information as possible from both sides of the drug and target. The exploration content expanded from intra-class to inter-class. Meanwhile, as the research on AI methods for predicting DTI has gradually deepened, researchers have begun to enrich the content of model inputs by incorporating more sources of information into the model; in addition to the known relationship information between drug targets, they have also introduced the associated information of side effects, diseases, genes, etc., and have expanded the information from the sequence information to the molecular structure diagrams and three-dimensional models on both the drug and target sides to construct a model with multi-perspective information. On the drug and target sides, the information is also extended from sequence information to molecular structure maps, 3D models^17^, and other multi-perspective information to construct a larger and more robust multimodal heterogeneous prediction network. The processing method for these complex nodes is also gradually expanded from simple direct node relationship processing to the expansion of indirect nodes with more “contextual” relationships to form a network graph structure and find the meta-paths of inter-node pathways. For example, in 2020, Wang et al.^18^ constructed interaction edges for drugs, targets, diseases, and side effects and subsequently eliminated orphan nodes to create an extensive interaction network. The similarities between nodes considers both first and second order connections. Each node is viewed as both a vertex and the “context” of another node, enhancing the detection of interrelationships through neighborhood indirection. In 2021, Liao et al.^19^ contend that for prediction purposes, the utilization of atomic-level sequences, such as molecular graphs surpasses the use of character-level sequences such as SMILES strings in prediction.

#### Sample-data processing

Although the datasets are rich in types, originate from a vast range of sources, and have diverse modalities, they also have some issues with the actual training of the model.

1. The definition of negative samples was not standardized. Some current methods define a negative sample as one in which an unknown DTI relationship is directly trained as a negative sample, whereas in reality, drug-target relationships that are not excluded from wet experiments are likely to remain undetected simply due to hidden interactions.
2. The digital divide between positive and negative sample sizes. The small number of known DTIs relative to unknown drug-target relationships result in an extremely unbalanced dataset, which affects the effectiveness of the model training^20^.
3. Specific sample sizes were not sufficiently rich, noisy, and multidimensional. Although the overall dataset is already large, there are not enough samples for specific data needs, particularly when using deep learning models, where the sample size is not sufficiently large to reach the optimal performance point of the model. Moreover, these samples are typically high dimensional and contain noisy data.
4. Sample cold start problem^21^. It is difficult for a model to deal with drugs or targets that do not exist in the training set but appear in the test set.

Therefore, several sample-data adjustment methods have been proposed for DTI prediction.

1. Reduce the scope of the dataset using only the identified negative samples to construct positive and negative sample sets.
2. Re-split and merge the positive and negative sample pairs in the dataset, thus reducing the gap in the sample size.
3. Expanding the dataset and conducting data augmentation.
4. A new strategy was adopted to divide the training set into test sets.

The specific solutions are enlisted in Table 2.

**Table 2:** Data processing methods

Type of problem	Methodological characteristics	Author
Definition of negative samples is not standardized	High-quality negative samples are selected through the screening process to reconstruct the sample dataset, while the spherical search method is used to identify DTIs to avoid falling into local optimums and optimize the recognition ability of the extreme learning machine.	Hu et al.^69^
The digital divide between positive and negative sample sizes	An integrated learning framework for negative stacking that narrows the gap between positive and negative samples by sampling and splitting the negative samples, followed by integrated training.	Yang et al.^70^
	The data expansion method Synthetic Minority Oversampling Technique (SMOTE) was introduced to generate new samples from a small number of existing samples.	Calangian et al.^71^
Specific sample sizes are not rich enough and are noisy and multidimensional	A lightweight learning framework light deep convolutional neural network, LDCNN-DTI, uses fewer protein descriptors and is able to convolve amino acid sequences of different lengths.	Wang et al.^18^
Cold start problems	Re-split the dataset. Split the positive samples into 5 groups, randomly select the negative samples as counterexamples, and combine them into 4 training sets and 1 test set.	Li et al.^72^
	An unsupervised approach is used to introduce both intra- and inter-class interaction information of drugs and targets into the prediction network using migration learning. This pre-training method also performs well on the DTA prediction task.	Nguyen et al. ^73^

## AI methods prediction process

### Traditional machine learning methods

Machine learning methods^22,23^ have long been important tools in drug discovery. DTI relationships are commonly represented by binary classification relationships; therefore, they are well-suited for some traditional classification algorithms. Traditional machine learning methods, including support vector machines^24,25^, random forests, naive Bayes^26^, and artificial neural networks^27^, are extensively used to model DTIs using quantitative structure-activity relationships, protein stoichiometry, and molecular docking methods. Support vector machine is a extensively used algorithm for classification issues that attempt to find a hyperplane to maximize the interval between positive and negative samples to achieve the classification task. Random forest and gradient boosted tree, an integrated approach, can deal with complex non-linear relationships and high-dimensional feature spaces and make classification predictions efficiently.

**Fig. 4:**
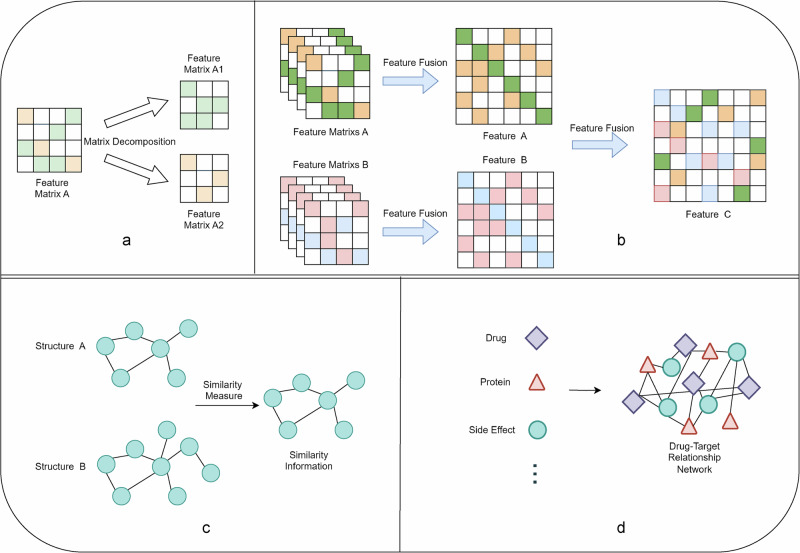
Four traditional machine learning methods. **a** matrix decomposition methods; **b** feature-based methods (feature fusion); **c** similarity-based methods; and (d) network-based methods.

Methods applied to DTI problems can be categorized according to the direction of problem solving into matrix decomposition^28^, similarity-based^29^, network-based^3^, and feature-based methods^30,31^, including programs that apply a mixture of these methods. The four foundation methods are depicted in Fig. 4. The matrix decomposition method is to represent the problem space as a matrix, and then the matrix is downsized and interpreted to extract the key features, and the common matrix decomposition methods include Principal Component Analysis (PCA), Singular Value Decomposition (SVD), and so on. There are two ideas for similarity-based methods: one is to consider the drug and target in the DTI problem as two different attributes, looking for similar associations between the attributes, and then docking and integrating this similarity information to deal with it; the other is to deal with the drug and target as a whole, looking for the similarity information between these two different data types. With the development of research on similarity information, the similarity information-based approach is not limited to the drug-target relationship itself but extends to the relationship between drugs, targets, diseases, genes, phenotypes, side effects, and other modalities, and more robust similarity relationships are constructed through this increasingly large information ecology. Similarity-based approaches look for similarity relationships between objects, whereas network-based approaches build multiple data into a data-network pathway to jointly solve the prediction problem. Feature-based methods include feature selection, feature fusion, and feature-based methods, all of which often require a priori knowledge. Feature selection involves filtering out important data features and retaining them after data processing. Feature fusion works by combining multiple features from diverse sources or diverse types to obtain a more informative and discriminative feature representation. Feature fusion can be achieved in various ways, including simple weighted averaging, feature stacking, or cascading. Common feature fusion methods include weighted fusion, stacking fusion, and cascading fusion, including rotating random forest^32^ and cascading forest depth (CDF) models^33^. The multicore fusion method is also a special feature fusion method, which is a common method for solving DTI problems; it divides the problem into multiple subspaces and combines diverse kernel functions to process the subspaces to integrate different feature information. Multi-kernel fusion can fuse diverse kernel functions in a linear or non-linear manner to obtain a more expressive feature representation.

A comparison of the advantages and disadvantages of the diverse machine learning methods is depicted in Table 3.

**Table 3:** Comparison of different machine learning methods

Approach	Advantages	Possible problems
Matrix Decomposition	Dimensionality reduction to reduce computational and storage overhead; noise reduction to extract potential features of the data; concise representation	Dimensionality disaster; performance is affected when data is sparse and there are too many default values; easy to overfitting; data processing relies on a lot of a priori knowledge;
Similarity-based methods	Highly interpretable; robust; can be used for unsupervised learning; focuses on local information;	Dimensional catastrophe; lack of construction of global information, poor generalization; high computational and storage overhead; sensitive to feature selection and choice of distance metrics
Network-based approach	Strong ability to capture interactions and contextual relationships; high interpretability; high generalization ability;	High computational complexity of graph structure; poor robustness to noisy data; sensitive to model parameters;
Feature-based approach	High noise immunity; high ability to synthesize and extract information; high ability to transfer learning across domains and modalities; high flexibility in strategy adjustment;	High computational complexity; high parameter sensitivity; high data distribution sensitivity; high a priori knowledge requirements

With the continuous development of traditional machine learning methods, an increasing number of research approaches have combined the above research directions to address the DTI prediction problem. For example, Liu et al.^34^ argued that some past methods based on similarity integration either do not consider the global interaction information at all or only consider the global interaction information, ignoring the importance of structure and association topology. Therefore, they proposed a similarity integration method based on Local Interaction Consistency (LIC) for preprocessing data to construct similarity matrices, and three types of matrix decompositions were used to optimize the AUPR and AUC metrics.

The specific machine learning methods are described in Table 4.

**Table 4:** Introduction to specific methods of machine learning

Author	Year	Approach	Method description	Characteristic
Jamal et al.^31^	2021	Matrix decomposition	A non-negative matrix tri-factorization method that is able to connect the bases of the drug-target space by a special matrix X without forcing alignment by pre-decimation or discarding.	This method has a few more degrees of freedom than the traditional two-factorization method.
Ye et al.^74^	2021	Matrix decomposition	A graph regularization method that introduces non-negative constraints and non-trivial solution constraints to constrain negative values in matrix values to optimize low-dimensional matrices containing potential correlators to predict drug-target interactions. The focus is on capturing the internal structural information of drug-target data for representation learning.	Negative values usually have no practical significance (either there is a relationship and the value is >0, or there is no relationship and the value is equal to 0, so negative values are usually meaningless).
Ahn et al.^75^	2022	Similarity-based methods	Based on similarity Applying Kullback-Leibler scattering to the prediction of DTIs, a random forest model was developed by using 3D molecular fingerprints of drugs as a representation of drug molecules to do similarity estimation of targets and ligands.	This model is able to extract the similarity information of drug-target pairs from the chemical similarity level.
Zhou et al.^3^	2020	Network-based approach	Network-based ranking algorithms to predict DTI by integrating large multimodal data networks of drugs, targets, diseases, genes and genetic relationships .	Covers multiple modalities with rich data content and network robustness.
Ozsert et al.^76^	2023	Feature-based approach	Feature selection is done by using autoencoder as a symmetric learning method for SMILES for capturing similarity based binding between receptor and ligand.	Combining feature selection with similarity-based methods performs better at the level of interpretability.
Liu et al.^34^	2021	Similarity-based method, matrix decomposition	An approach based on similarity methods and matrix decomposition uses a similarity integration method based on local interaction consistency (LIC) to preprocess data to construct a similarity matrix, and then three matrix decompositions are used to optimize the AUPR, AUC metrics.	The similarity integration is used to capture local structural information, and the matrix decomposition is used to target and optimize the evaluation metrics.
Liu et al.^77^	2022	Matrix decomposition, similarity-based method	A method combining matrix decomposition and random walk strategy (MDMF) models multiple similarity integrated structures with high order similarity.	It breaks the limitations of matrix decomposition methods, which are prone to dimensional catastrophe and overfitting, and random walk methods, which are directionless and slow to converge.

### Deep learning methods

As deep learning methods have become increasingly mature, existing research has gradually shifted from machine learning to deep learning methods. Khan et al.^35^ found that the random under sampling method affects model performance by comparing diverse machine learning and deep learning methods, and the performance of deep learning with diverse machine learning methods applying dataset resampling techniques compared to deep learning without resampling techniques is outstanding.

In the field of predicting drug-target interaction relationships, a number of deep learning methods are extensively used, including sequence model-based methods, graph neural network-based methods, deep generative learning-based methods, and incorporating attentional mechanisms into these methods, which allow the model to focus more on finding specific components and patterns in some data. For example, researchers have been widely interested in the transformer since it was proposed as an idea, an approach as vivid as the name of its paper, “Attention is All You Need”. The attention mechanism has been very popular in the last two years, and applied research based on the transformer method is very popular. The possibilities of improvement from diverse data processing stages including constructing some kind of linkage among different modules and objects are extensively discussed and experimented upon by researchers. Drugs and targets are two different objects that need to be connected between them, and perhaps an impressive connection can be found in the black-box model of deep learning.

#### Sequence model-based approaches

**Fig. 5:**
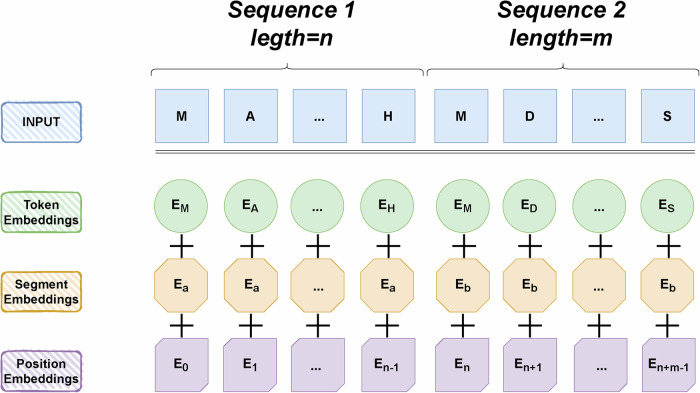
Common sequence-based language modeling architectures: Bert^68^ as an example. Bert’s model, in order from top to bottom, encodes the input sequence into textual and positional information to obtain absolute and relative positional information. Then masked language modeling is performed to predict the next sentence to handle the sentence-level classification task.

For the prediction of protein sequences and drug molecular structures, sequence-model-based approaches, including recurrent neural networks or long- and short-term memory networks can be used to capture temporal dependencies in sequences. Jin et al.^36^ proposed the EmbedDTI approach to predict drug-target interactions by enhancing molecular expression through sequence embedding and graph convolutional networks. For protein sequences, feature embeddings of amino acids are pre-trained using language modeling and fed into a convolutional neural network model for representation learning. A typical sequence-based language model architecture is depicted in Fig. 5. Liu et al.^37^ (2022) applied the Transformer method to drug-target interaction (DTI) recognition. Their study demonstrates that Transformer can effectively extract deep features even when only sequence information is used. Huang et al.^38^ (2022) introduced the CoaDTI framework, which applies pre-trained encoders to protein encoding to address the issue of sparse labeled data. It is a Transformer-based model with a co-attention mechanism, extracting required features from both original protein sequences and drug molecule SMILES. Under transfer learning and pre-training guidance, CoaDTI significantly improves performance and shows good generalization capabilities on identifying new DTIs.

Methods based on sequence models not only extract intrinsic features of data but also consider the correlations between data contexts. Particularly, chemical structures like drugs and proteins may contain complex long-range contextual information such as chemical bond connections, cyclic structures, and polarity relationships. Therefore, the ability to learn such contextual information is crucial for handling sequence data. Zheng et al.^39^ (2023), building upon graph neural networks, employ a dual participation of multi-head attention mechanisms to identify critical binding features in sequences, thus partially addressing long-range dependencies. Zhao et al.^40^ (2022) proposed the GIFDTI model for long-distance encoding, splitting drug, target, and interaction information into three modules to extract key features. Integrating CNN and Transformer in the module for local feature extraction captures and encodes features in long distances, combining global information, local features, and inter-class correlations. This model better learns local pattern information in sequences.

In contrast to context-independent methods like word2vec, BERT^41^ (2019) is a context-based language model. Its introduction prompted exploration into fine-tuning models based on language model pre-training, leveraging its powerful inference capabilities for tasks such as drug discovery. Kang et al.^42^ (2022) utilized ProtBert, a BERT-based pre-trained model for amino acids, and RoBERTa pre-trained for encoding drug SMILES format, significantly enhancing performance in affinity prediction tasks.

#### Methods based on graph neural networks

These are a class of deep learning models that specialize in graph-structured data. In predicting drug-target interactions, drugs and proteins can be represented as graph structures, and then the nodes and edges of the graph can be represented by learning using Graph Neural Network(GNN) to predict their interactions. In traditional network analysis, only direct interactions are considered, neglecting local effects of nodes (such as neighbors and edge direction) and global positional information (like global topology or structure). Graph Neural Networks (GNNs) sample and aggregate features from local neighbors, preserving both the local roles of nodes and global positional information within the graph.

**Fig. 6:**
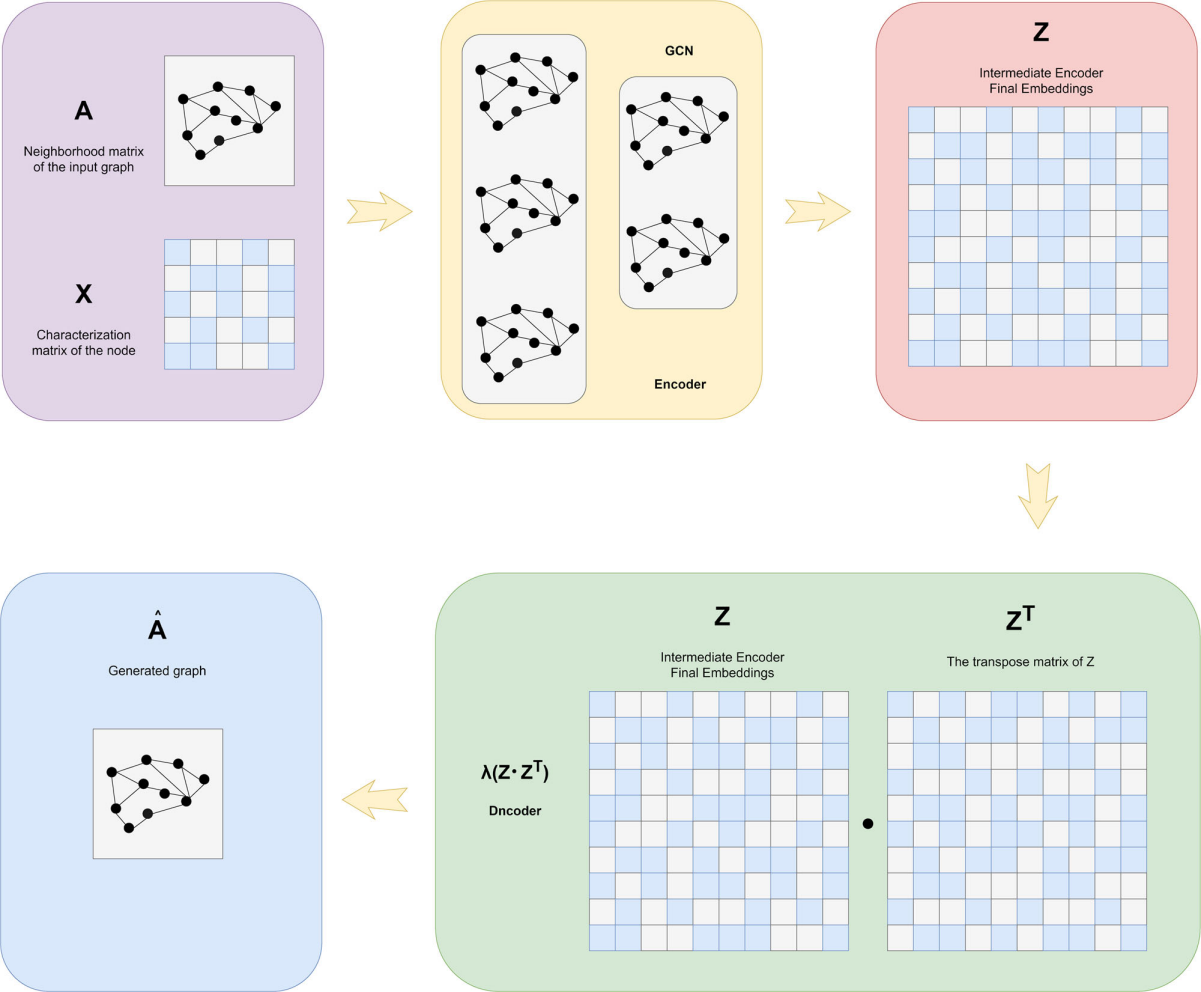
A basic variogram auto-encoder (VGAE). The input graph’s adjacency matrix A and the node’s identity matrix X are used to learn the mean *μ* and variance *σ* of the node’s low-dimensional vector representation through an encoder (graph convolutional network), which is then used to generate the graph with a decoder (link prediction).

In 2020, Yin et al.^43^ proposed the DeepDrugw method, a general deep learning framework for predicting drug-drug interactions (DDIs) and drug-target interactions based on graphs. It utilizes Residual Graph Convolutional Networks (RGCNs) and Convolutional Networks (CNNs) to learn structures and sequences of drugs and proteins, improving prediction accuracy of DDIs and DTIs (Drug-Target Interactions). In 2021, Peng et al.^44^ introduced an end-to-end heterogeneous graph learning architecture EEG-DTI, which incorporates multiple modalities of biological information as inputs and learns low-dimensional feature representations of data end-to-end during model training. In 2022, Li et al.^45^ observed that previous methods often overlooked integrating node attributes and relationships comprehensively, typically focusing on only one aspect during processing. Therefore, Li et al. proposed the GCNDTI method, utilizing graph neural networks to integrate node attributes and topological information, resulting in more representative low-dimensional features. Qu et al.^46^ proposed the Graph-DTI approach, which is a new model for drug-target interaction prediction based on heterogeneous network graph embeddings, by using a GCN-inspired graph autoencoder to extract higher-order structural information to learn the nodes (drugs, proteins) and their topological neighborhood representations to form a heterogeneous network. Yu et al.^47^ proposed a heterogeneous graph neural network model based on Bi-LSTM and an attention mechanism to enhance the node feature acquisition and heterogeneous data aggregation, while a negative sampling technique was used to further optimize the predictive ability of the model. A typical graph neural network approach is depicted in Fig. 6.

#### Methods based on deep generative models

Deep generative models based on GAI, are a type of AI that generates new representations by learning from existing data. These new representations can resemble existing data or be improved versions, spanning various types such as text, speech, video, and more. Their emergence has further advanced the concept of designing drug molecules from scratch. Unlike other methods that rely on learning molecular representations from vast datasets for pattern recognition and prediction, this approach not only identifies drugs with similar effects but also potentially begins with mimicking and optimizing local small molecules to design drug molecular configurations, gradually adapting to the construction of larger molecules^48^. It supplements data from a learning-generating-discriminating perspective to enhance the model’s robustness.

**Fig. 7:**
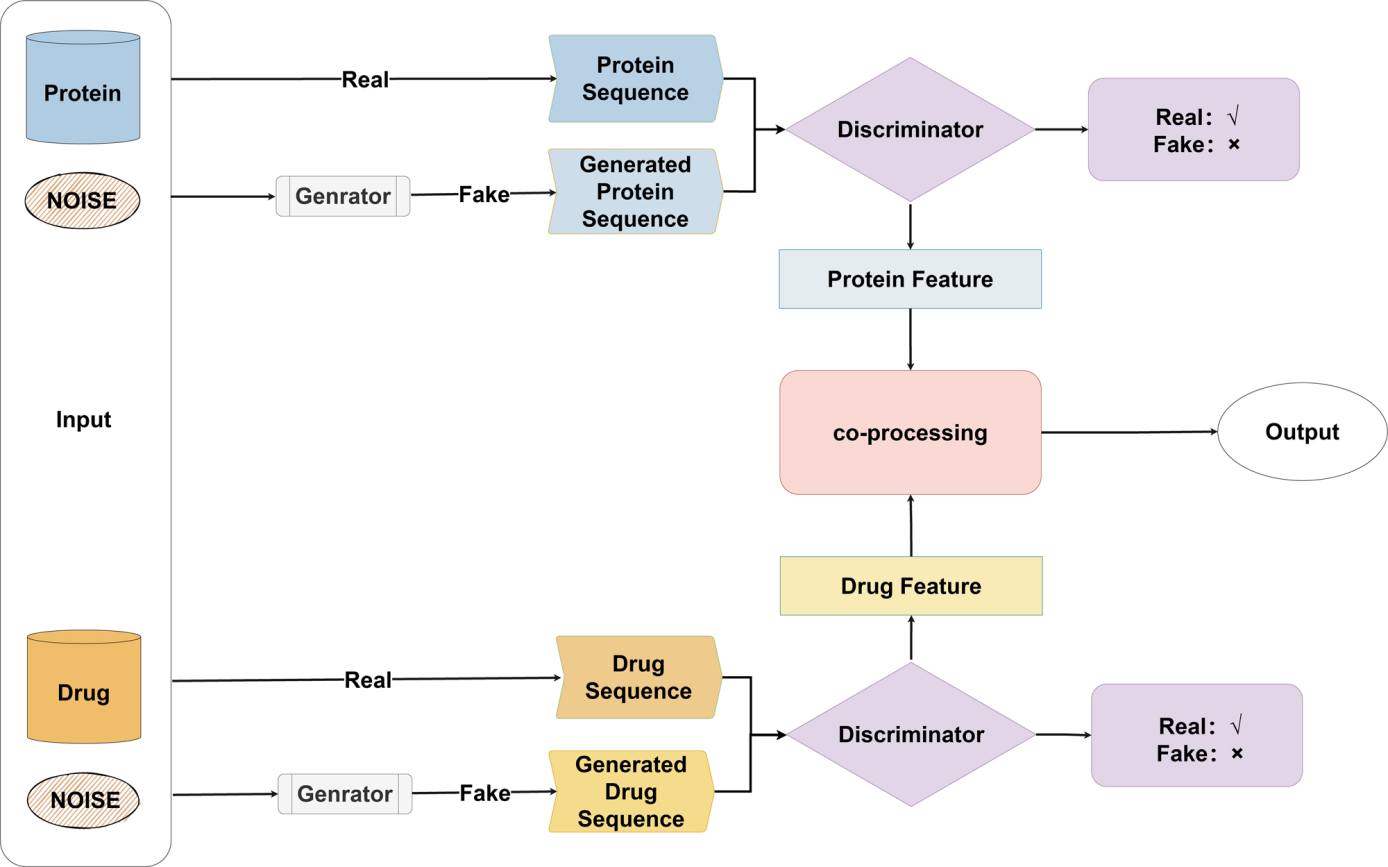
DTI prediction using generative adversarial networks. Both sides of the drug and target put raw and noisy data into the inputs, process the noisy data into false data through the generator, and then put both true and false data into the discriminator to determine the right and wrong.

These models can learn data distributions and generate new samples. In predicting drug-target interactions, deep generative models, including variational autocoders or generative adversarial networks or diffusion models^49^, can be used to generate new interactions by learning potential representations of drugs and proteins. Zhao et al.^50^ proposed the GANsDTA approach to predict binding affinity based on semi-supervised generative adversarial networks (GANs), which comprises two parts: two GANs for feature extraction and a regression network for prediction. This is the first semi-supervised GAN-based method to predict binding affinity. A GAN with noisy data added for DTI prediction is depicted in Fig. 7.

In 2022, attention was focused on the reinforcing effects of attentional perception and transfer learning on GAI in drug discovery. Li et al.^51^ proposed the HGAN method, which constructs a heterogeneous graph attention network with an added graph attention diffusion layer. This method captures more indirect node information from both intra- and inter-layer perspectives, enlarging the receptive field and enhancing attention perception. The study also found that varying the degree of receptive field expansion within and between layers leads to different effects. Specifically, when the receptive field is expanded to the same extent, inter-layer expansion achieves better model prediction performance compared to intra-layer expansion. Wang et al.^52^ introduced the GCHN-DTI method, a graph convolutional approach based on heterogeneous networks for predicting drug-target interactions. GCHN-DTI integrates network information including drug-target interactions, drug-drug interactions, drug similarity, target-target interactions, and target similarity. Graph convolution operations are performed within the heterogeneous network to obtain embeddings for drugs and targets. Additionally, an attention mechanism is introduced between graph convolution layers to jointly process node embeddings from each layer. Finally, drug-target interaction scores are predicted based on the embeddings of drugs and targets. This model uses fewer network types and demonstrates higher predictive performance. Nguyen et al.^53^ faced with insufficiently rich coronavirus datasets, enhanced data atomically and proposed a graph neural network method. They introduced a multi-hop gating mechanism to improve model training by obtaining better atomic representations from non-neighboring information. The authors suggested that incorporating large datasets for transfer learning could further enhance model performance.

In 2023, there was a renewed interest in the impact of self-encoder quality on the performance of the underlying model, and a focus on the ability of metapath to semantically capture and denoise the generative model, optimizing the generative learning model in terms of the underlying path of the model. Zhang et al.^54^ infer features for the unknown drug-target space and propagate labels for known DTIs using two different graph autoencoders to form an enhanced deep autoencoder. This approach aims to extract latent information in such heterogeneous topologies. Li et al.^55^ proposed the MHTAN-DTI method, which, compared to other GNN-based approaches, enhances the capture of higher-order structural and semantic information in biological heterogeneous graphs through meta-path expansion. This method leverages a hierarchical transformer structure to model contextual information, assigns weights to different meta-paths, introduces an attention network for weighted fusion, distinguishes the importance of different meta-paths, reduces the impact of noisy data on the model, and improves model robustness. Chen et al. introduced the SDGAE method, a graph convolutional autoencoder approach^56^. Through preprocessing steps, SDGAE embeds nearest-neighbor relationships among nodes while preserving the graph structure to maintain the topological relationships during graph-based learning. This approach effectively addresses the issue of numerous isolated nodes in traditional graph convolutional autoencoder models.

#### Mixed methods and multimodal modeling

**Fig. 8:**
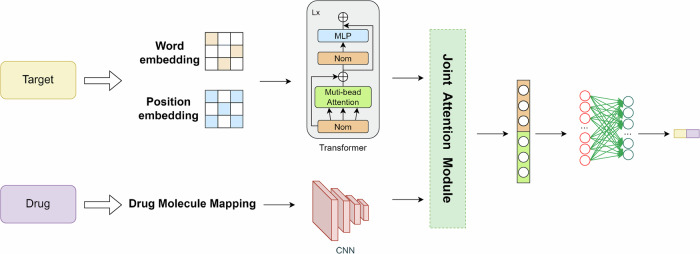
Introducing joint attention mechanism for DTI prediction network based on transformer and CNN dual tower structure. In the prediction process, convolutional neural networks are employed on the drug side to process molecular graphs, while Transformer models are utilized on the target side for handling word embeddings and position embeddings. Subsequently, the data processed through the dual-tower model is integrated into the joint attention mechanism for further refinement and organization of key features.

**Fig. 9:**
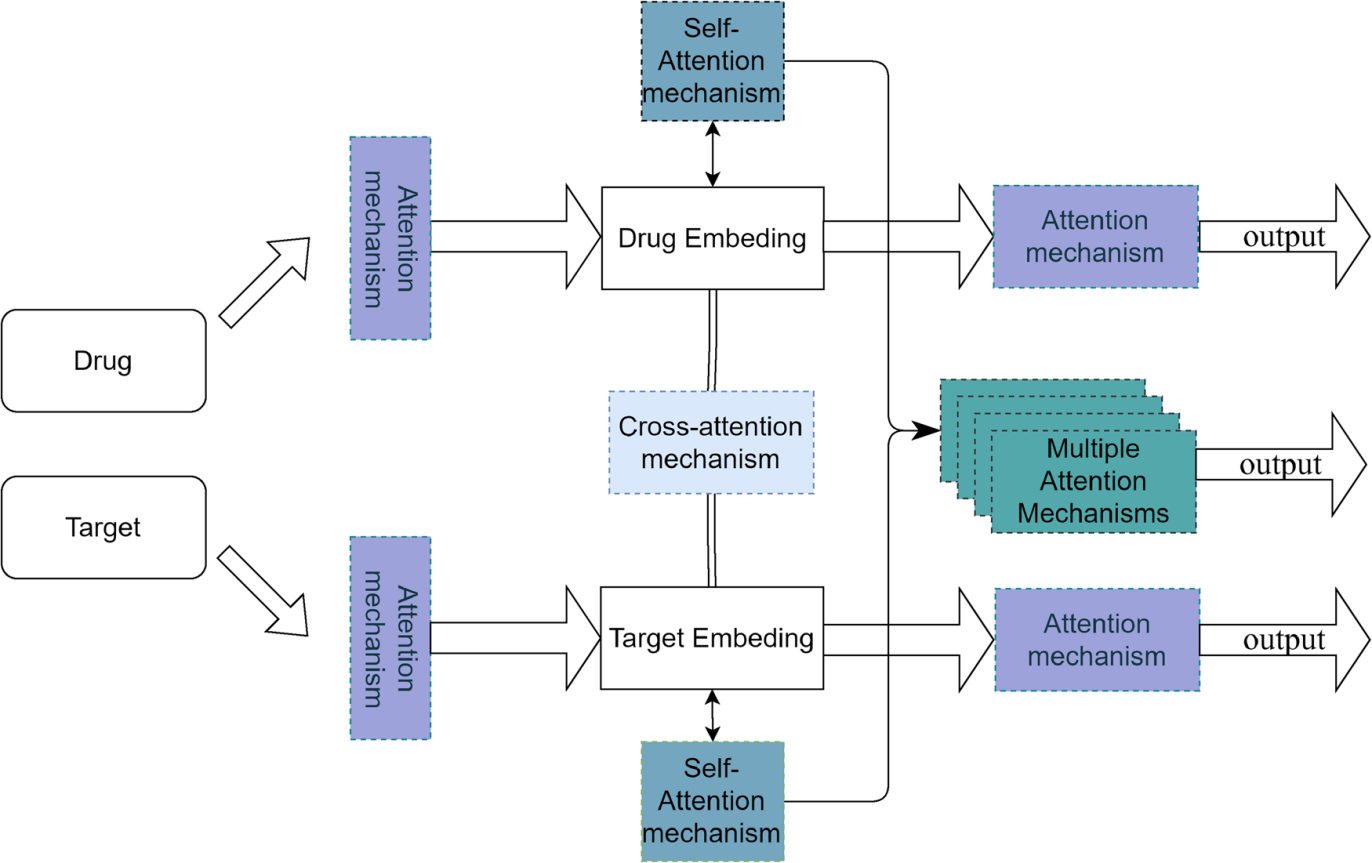
Common Scenarios for Using Attention Mechanisms. The single-head attention mechanism, the cross-attention mechanism, the hybrid attention mechanism, and the multi-head attention mechanism are inserted at various locations of the model in the figure, referring to the fact that attention mechanisms can be added to various modules and stages of the model to enhance the feature capturing capability of the model.

With the gradual development of cross-modal and multimodal models, an increasing number of sequence-based language models and graph representation learning models based on graph neural networks have been combined to completely combine the features of the two representations in the prediction of proteins and targets and more comprehensively achieve the modeling of connective intuition for interpretability. A typical DTI prediction network based on the twin-tower structure of Transformer and CNN is depicted in Fig. 8. Meanwhile the development of deep generative learning networks has enriched the horizon of DTI prediction from the perspective of ab initio construction. Researchers have also explored the attention mechanism from different perspectives, including the single-head, multi-head, joint, and cross-attention mechanism, to find the key points of model construction and the key links of data interpretation. Common scenarios using the attention mechanism are depicted in Fig. 9.

Our Table 5 chronologically documents a number of deep learning methods, including the author, year of publication, dataset used, method-specific description, and method characteristics.

**Table 5:** Introduction to Deep Learning Methods

Author	Year	Dataset	Method description	Characteristic
Zhao et al.^78^	2020	DrugBank	Improved graph representation learning with multiple data.	The information related to drugs, targets and drug-target pairs are merged into the heterogeneous graph. This method enriches the meaning of node information and optimizes the excessive smoothing phenomenon in traditional graph neural networks.
Monteiro et al.^79^	2020	Private dataset	Based on Convolutional Neural Network (CNN) with inputs as protein sequences and structured compounds SMILES.	This approach is preferable to obtaining a table of data from a traditional descriptor.
Zhao et al.^50^	2020	Davis, KIBA	A semi-supervised approach based on Generative Adversarial Networks (GANs) for predicting binding affinities.	The method consists of two parts, two GANs for feature extraction and a regression network for prediction, which is the first method based on semi-supervised GANs to predict binding affinity.
Jin et al.^36^	2021	KIBA	Enhanced molecular expression prediction of drug-target interactions by sequence embedding and graph convolutional networks.	For protein sequences, feature embeddings of amino acids are pre-trained using language modeling and fed into a convolutional neural network model for representation learning.
Wang et al.^80^	2021	DUD-E, human, BindingDB	Multitasking neural network consisting of a graph neural network, an attention module, and a multilayer perceptron.	Good multitasking performance, excellent in both binding affinity prediction and interaction categorization.
Huang et al.^81^	2021	Biosnap, Davis, BindingDB	The method specifically selects a knowledge-heuristic pattern mining algorithm for the substructures and models the interaction of the data on this basis. An enhanced transformer encoder is also employed in unlabeled data to capture information about its substructures.	Fully exploiting parsing of substructures and potential knowledge of unlabeled data.
Cheng et al.^82^	2021	human, C.elegans, DUD-E, DrugBank	Based on graph neural networks, the key binding features of sequences are searched for through two engagements of the multiple attention mechanism.	Convolutional neural network is affected by the size of the convolutional window, and can only extract the local correlation information in the window area, but not the long-range contextual relationship. This method can solve the long-range dependency problem of sequences to some extent.
Peng et al.^44^	2021	Luo and Yamanishi	An end-to-end heterogeneous graph learning architecture.	The input to this heterogeneous network contains biological information in multiple modalities, and a low-dimensional feature representation of the data is learned end-to-end in model training.
Wang et al.^83^	2022	Private dataset	Construction of a knowledge graph of drug-target pairs.	Knowledge graph to get recommendation information.
Li et al.^45^	2022	Luo	Graph neural networks are utilized to synthesize node attributes and topological information.	The extracted low-dimensional features are more representative, taking into account the nodes’ own attributes and the relationships between nodes.
Yu et al.^47^	2022	Luo	A framework for predicting drug-target interactions by information aggregation based on heterogeneous graphical neural networks with incorporation of attention mechanisms.	The method first obtains the molecular fingerprint information of the drug and the pseudo-amino acid composition information of the protein, and then extracts the initial features of the nodes by Bi-LSTM and aggregates the heterogeneous neighbors using the attention mechanism.
Wang et al.^52^	2022	Luo	A method for predicting drug-target interactions based on the convolution of heterogeneous network graphs integrating network information on drug-target interactions, drug-drug interactions, drug-drug similarity, target-target interactions, and target-target similarity.	Graph convolution operations are performed in the heterogeneous network to obtain node embeddings of drugs and targets. An attention mechanism is also introduced between the graph convolution layers to jointly process the node embeddings from each layer. The model uses fewer network types and has high predictive performance.
Nguyen et al.^53^	2022	PDBbind, Fragalysis	A method based on graph neural networks that introduces a multi-hop gating mechanism, as well as an atomic enhancement design for data in the face of insufficiently rich coronavirus datasets.	Better atomic representations can be obtained from non-neighbor information to improve the training effect of the model, while the authors mentioned that if a large dataset can be introduced to do migration learning can improve the model performance to better.
Zhao et al.^84^	2022	Kinase, Davis, Metz, KIBA	Deep sequence learning for drug target binding affinity prediction based on attention mechanisms.	Leverage attention mechanisms to focus on important key subsequences in drug and protein sequences to predict affinity.
Zhang et al.^4^	2022	Tang, DrugBank, KEGG and PubChem	Transformer-based deep learning model with convolutional network and graph neural network can effectively extract the local residual information in the sequence, and the interaction data of molecular structure.	
Wang et al.^85^	2022	BindingDB	A multi-view strategy with converters is used to achieve dimensionality reduction for matrix dimensions, while an end-to-end model is used to reduce the complexity of the model with feature extraction steps.	Simplifies the characterization learning task for the acquisition of locally important residues.
Huang et al.^38^	2022	human, C.elegans, BindingDB	A Transformer-based shared attention mechanism model for extracting desired features from raw protein sequences and drug molecule SMILEs, respectively.	Apply pre-trained encoders to protein coding to solve the problem of scarce labeling data. And introduce migration learning as a pre-training guide.
Zhao et al.^40^	2022	DrugBank, Davis, KIBA, BindingDB	In the module used for local feature extraction of sequences, CNN and Transformer are combined to be able to capture and encode feature information over long distances, and then finally unite the global information with the local features as well as inter-class correlation information.	By dividing the drug, target and interaction information into three modules and extracting the key features separately, the local pattern information in the sequence can be better learned.
Wang et al.^86^	2022	human, C.elegans, Davis	Combines graph convolutional neural networks and transformer methods with a multi-head attention mechanism to focus on atomic structure information.	The ability to learn conformationally stable atomic information demonstrates its interpretability at the atomic level.
Li et al.^87^	2022	human, DUD-E, BindingDB	A bipartite interaction messaging module has been used to capture the bidirectional effects of drug-target interactions, and the weight display has been optimized for better interpretability.	The importance of focusing on both sides of the drug and the target.
Ranjan et al.^88^	2022	MOSES	Combining a graph neural network approach with a knowledge graph approach and using an early fusion approach to representational screening of protein tertiary structure and order predicts the binding affinity scores of the generated molecules.	This combined approach effectively reduces the number of generated molecules and retains most of the theoretically well-bound molecules.
Liu et al.^37^	2022	DUD-E, LIT-PCBA	An intermolecular map conversion method that incorporates a special attention mechanism used to find topological and spatial information between molecules, and uses a three-way transformer to model intermolecular information.	This approach excels at predicting binding positions and has been validated for drug screening of coronaviruses.
Ye et al.^89^	2022	the gold standard dataset, DrugBank	A graph autoencoder and multisubspace deep neural network.	Enhancing its learning capabilities through automatic coding of graphs, subspace layers, and integration layers allows for more features to be extracted from network inputs and for better training of DNN networks.
Bae et al.^90^	2022	Davis, BindingDB	A DTA prediction model that can take into account local substructures. It incorporates an attention mechanism between drugs and proteins that focuses on local information while focusing on global information.	Attention has been given to the interrelationships between protein and drug substructures, and it is thought that it is the interaction between certain specific substructures that is critical for the binding of these drugs and targets.
Joshy et al.^91^	2022	KIBA	Based on CNN, the inputs are the FASTA of the protein and the neural fingerprint of the drug, and a multilayer interaction network of the two is constructed to realize the prediction of DTA.	This approach effectively realizes the embedding of drug molecule structure by focusing on chemical properties such as drug molecule structure during drug data processing.
Mehdi et al.^92^	2022	DUD-E, Human, BindingDB	A graph deep learning model that incorporates an attention mechanism tries to understand this internal semantic-level association and external intermolecular association of drugs and targets from an NLP perspective.	Interpretation of drug-target associativity relationships as sentence-level relationships. Stronger interpretability in the context of applying NLP methods.
Wu et al.^93^	2022	Drugbank, KEGG	A knowledge graph attention network that decomposes the representation of relationships between nodes with a knowledge graph and adds an attention weighting scheme to filter out important features.	The method cleverly converts the DTIs classification prediction problem into a neighborhood node linkage prediction problem for knowledge graphs.
Li et al.^51^	2022	Hetero-A, Hetero-B	A heterogeneous graph attention network with an additional graph attention diffusion layer.	More indirect node information is captured in the intra-layer and inter-layer perspectives, thus expanding the perceptual field and realizing attentional perception enhancement. The method also found that the effect of expanding the perceptual field is different to different degrees in the intra-layer and inter-layer perspectives, and when the perceptual field is expanded to the same degree, the inter-layer perceptual field can be expanded to achieve better model prediction than the intra-layer perceptual field.
Wang et al.^94^	2022	Davis, Biosnap, DrugBank	A parallel decoupling method combining CNN and Transformer.	Local and global features are extracted separately and coupled by a cross-attention mechanism, while the interaction information of drug-target pairs is convolved with a dynamically generated filter to achieve a better representation for local and global features.
Qu et al.^46^	2022	DrugBank, HPRD	A new model for drug-target interaction prediction based on heterogeneous network graph embedding.	Learning nodes (drugs, proteins) and their topological neighborhood representations by extracting higher-order structural information using a GCN-inspired graph autoencoder to form heterogeneous networks.
Ma et al.^95^	2022	PubChem, DrugBank, UniProt	Adding multiple self-attention mechanisms to both drug processing and drug-target co-processing phases.	Multiple attention mechanisms can help improve the model’s handling of specific requirements.
Yin et al.^43^	2023	DrugBank, TwoSides dataset, DDInter	A generalized deep learning framework for graph-based prediction of drug interactions and drug target interactions.	Learning the structures and sequences of drugs and proteins using Residual Graph Convolutional Networks (RGCNs) and Convolutional Networks (CNNs) to improve the prediction accuracy of Drug-Drug Interaction (DDI) and DTI.
Zhang et al.^54^	2023	DrugBank, UniProt, MalaCar	Two different graph autoencoders are used to form an enhanced deep autoencoder.	Feature inference and label propagation between known DTIs for spaces containing unknown drug targets.
Bian et al.^96^	2023	Davis, KIBA, DrugBank	A multiple cross-attention mechanism based on weight sharing is employed, using only sequence information as input.	The 2D feature maps of proteins and drugs are compressed into 1D feature maps, which are then connected and fed into FCN for classification. It can effectively solve the class imbalance problem and overfitting problem in drug-target datasets.
Wen et al.^97^	2023	Human, C.elegans, GPCR, Davis	Extraction of long-range interdependent features of sequences using a multi-attention mechanism to extract atom-amino acid interaction features.	Focus on the complex interactions between internal atoms and macromolecular compounds.
Kavipriya et al.^98^	2023	KEGG BRITE, BRENDA, SuperTarget, DrugBank	An optimal recurrent neural network-based approach using a semi-supervised approach based on BiLSTM model of RNN to handle inter-drug and inter-target interactions and initiated as weights.	Since the performance of the BiLSTM technique is strongly influenced by the hyperparameters, the Adam optimizer is used to adjust the hyperparameters during the model training process to improve the prediction performance.
Zheng et al.^39^	2023	DrugBank, BindingDB	A multi-task learning mechanism with two transformer encoder-decoder structures is introduced to enhance the extraction of target features.	The first to apply Mol2Vec, BERT, attentional mechanisms and multitasking mechanisms to a single model.
Zhu et al.^99^	2023	Davis, KIBA	Sequence features and substructural features of individual molecules are extracted by the self-coder to express relationships, while information interaction paths are established between molecules and sequences, forming an associative learning mechanism.	The correlation between substructures is better expressed, which improves the accuracy of the predicted values for the DTA task.
Sofia et al.^100^	2023	Davis, KIBA	Based on CNN, inputs are in SMILES and protein residue information.	There is no need to construct a similarity matrix, thus improving computational efficiency compared to some machine learning models.
Chen et al.^56^	2023	DrugBank, HPRD, CTD, SIDER	A graph representation learning method that embeds nearest neighbor relationships between nodes to keep the topology of the relationships between nodes undeformed in the graphical learning process while maintaining the graph structure.	Effectively solves the problem of large number of isolated nodes in traditional graph convolution autoencoder models.

### Cross-domain learning of DTI methods

As DTI problems are solved in-depth, several directions inevitably step into a bottleneck. These problems include insufficient integrity of data set, high dimension of data, noise and poor reliability of data. The running speed is slow, and the hardware equipment needed to improve the computing power is expensive; The model itself also has limitations, such as poor parallel ability of the algorithm, poor interpretability, long parameter optimization time, etc., and poor generalization ability of the model. A large amount of data is needed to achieve the best effect of the model.

Therefore, learning mature solution ideas from other domains can provide positive feedback on the improvement of modeling capabilities. In the problem of interaction prediction of drug-target pairs, for example, a common solution is to compare the molecular sequence data of the drug and the target, then extracting information from such sequence data can easily be associated with the field of Natural Language Processing (NLP), so there are a lot of researchers who divide the description language data of the drug-target molecules for processing from the NLP perspective.

Some common recommender system methods are also introduced into DTIs prediction: collaborative filtering methods can recommend targets according to similarity, and SVD can conduct data dimensionality reduction and noise reduction processing. Zheng et al.^57^ built multiple kernels on heterogeneous data from diverse information sources, and then proposed a joint method using collaborative filtering and SVD, which performs dimensionality reduction and noise reduction processing on data in the space of multiple kernels before conducting an associative search for recommendation to categorize and sort the potential DTIs according to similarity ranking.

The DTI problem can also be extended to the computer vision (CV) domain depending on the type of data used. For example, by using a 3D structural visual representation of a drug target or reducing such a representation to a 2D level to conduct tasks, including similarity judgment, target detection, and segmentation on a 3D model or image, refinement problems, such as structural configuration and binding pose that may be encountered in DTIs prediction can be solved by methods in the CV domain.

## Challenges and opportunities

Most of the datasets used for prediction were unbalanced, the proportions of positive and negative samples were seriously imbalanced, and the judgment criteria for negative samples were not sufficiently accurate. As the modalities of using data become increasingly complex, a deeper understanding is required for the data representation description, alignment, and unification of multimodal data, including specialized and joint processing methods for different data categories. Traditional machine learning methods have gradually entered a bottleneck period, whereas deep learning can adapt to more complex and diverse data changes and capture deep potential knowledge. The amount of data in the DTI dataset was not sufficiently large for the deep learning network, and the training of the parameters was insufficient, making it difficult to achieve the best model performance. With the increasing number of open data sources, the channels for obtaining data resources are becoming wider, and data information is becoming increasingly abundant. Conventional methods use only a single data category for model prediction, whereas deep learning networks have gradually been able to handle deeper information. Therefore, to tap into the predictive potential of deep networks, more relevant information can be incorporated to the model to enrich the data categories, types, dimensions, and horizons, thereby improving the mining of implicit correlation information in heterogeneous data. However, some professional data still have issues with insufficient sample sizes. For example, there is still a large gap between the total number of proteins with known three-dimensional structures and the total number of proteins with known sequences. However, judging from the prediction results of models using three-dimensional structures, the prediction accuracy of models that introduce three-dimensional structures can still be significantly improved compared with simple sequence prediction. As cryo-EM can achieve higher precision, the capture speed of protein structures may gradually accelerate, and the total amount of structural data will continue to increase. Therefore, if we can make good use of the known structural data, we can quickly achieve high-quality predictions from the model when the data expands. However, deep learning also faces problems, including long model training times, excessive randomness in tuning parameters, and poor interpretability. Exploring the optimization of deep learning prediction models from the bottom is another possible development trend.

Simultaneously, including the corresponding use of the latest domain knowledge, it is essential to broaden the understanding and application of deep learning methods and other knowledge, including CV, NLP, and the latest large-language models. Migrating mature methods from other fields for DTI prediction can also accelerate our understanding and application of DTI. Currently, the momentum for large model development is strong. Several fields have begun to actively access large models to adjust their corresponding training parameters. Researchers in diverse fields have begun to focus on large models. Several researchers in the field of biology have studied large-scale question-and-answer models. For example, in the medical field, Hong Kong Chinese (Shenzhen) launched “Hua Tuo,” etc., this series of research has focused everyone’s attention on large-language model development. If the DTI prediction problem can be combined with the excellent reasoning and prediction capabilities of large-language models, the ability of the DTI prediction model may be significantly improved.

Therefore, future research may start from the data itself, integrate and align the collected data, standardize data representation, and improve the sample screening and construction division of the dataset. It is also possible to improve the data fusion processing capabilities of the model from the perspective of the joint construction of multimodal models. Simultaneously, breakthrough innovations may occur when large models collide.

### Prospects for the application of LLMs

Since the advent of ChatGPT, the development of large language models has entered a period of high tide^58^. People are beginning to contemplate the role of LLMs in predicting drug-target interactions. Text sequence-based prediction tasks have been steadily advancing, but the integration of large models into everyday tools inevitably brings disruptive reforms to sequence-based prediction methods. One of the most fundamental changes we can think of is the reliability of predictions in context reasoning tasks, which were previously viewed with skepticism. The intelligent reasoning and response capabilities now exhibited by large models have significantly bolstered our confidence. Consequently, many are now attempting to provide large models with bioinformatics-related corpora, including sequence information on drugs and targets, structural details at the quantum chemistry level, protein folding information such as that provided by AlphaFold’s 3D structures, and external interaction data such as drug side effects, clinical characterization data, genetic information, and inheritance data across multiple dimensions and aspects. While handling such vast amounts of information posed a formidable challenge for individual prediction tasks, for large models, richer corpora yield more accurate reasoning outcomes. Additionally, we can develop intelligent integrated question-answering systems that infer predictions from limited user inputs, encompassing simple medical knowledge queries and complex structural inference queries. At the same time, methods based on large models face notable challenges, such as insufficient corpora, difficulties in data alignment, lengthy single training times, high trial-and-error costs due to uncertainties in parameter tuning, and demanding hardware requirements. However, it is clear that now is an opportune moment to advance drug-target interaction prediction based on large models.

### Some other concerns about AI approach

1. Deconstruction of data inputs: On the dimension of the data itself, researchers have focused on different methods of deconstructing sequences and on the impact of diverse classes of input information on prediction performance^59,60^. For example, Nandhini et al.^61^ used frequent subsequences in molecular representation learning to transform a raw dataset of drug targets into explicit substructures and form context-bound interactions.
2. Impact of data raw material: choice of macroscopic characterization observations or microscopic particle-level data inputs? For the dimensions of model construction and performance evaluation of prediction methods^62^, researchers have discussed how to classify prediction methods and optimize the performance evaluation, and Hinnerichs et al.^63^ classified the DTI prediction methods into the “top-down” method, which indirectly computes from observable features as raw materials, and the “bottom-up” method, which directly computes from information about molecular structures or sequences. Moon et al.^64^ proposed two key enhancement strategies to address the underperformance of the generalization ability of deep learning models by introducing the information equation of physics to capture the energy component of the interaction force of atom-atom pairs at the four levels and enhancing the generalization ability through data enhancement to improve the generalization capability.
3. Return to single drug analysis: the assessment of the predictive effectiveness of the model ultimately comes down to the predictive accuracy of a single drug. Grounding the prediction of DTIs to the level of a single-drug application is also a focus of DTIs research. Das et al.^65^ investigated the prediction of drug resistance against the virus by evaluating the performance of the HIV virus after the drug was introduced into the body through the GDL method. This study will be helpful in the performance evaluation of drug effectiveness post-development.
4. Data privacy and security issues: The data sources used for DTI prediction are extensively distributed, but the data involved in either experimental data on DTI, publicly available data sources, or investigative data, such as patients’ clinical data, require us to completely consider data privacy issues. Gianluca et al.^66^ discussed data privacy issues in the field of DTIs prediction. Compared to other domains, the drug-target interaction domain can be modeled with good privacy and security without compromising model performance. The study by Dániel et al.^67^ on the task heterogeneity of heterogeneous data also demonstrates that the application of a unified and standardized platform for managing private data in the DTI prediction domain has an acceptable impact on prediction performance. Therefore, effective measures can be taken to address public concerns about data privacy and security in the face of the push for more data.

## Conclusion and prospect

Issues and bottlenecks in AI-based DTI prediction include:

1. Data issues: Limited data samples, imbalanced positive and negative samples; high-dimensional and noisy data; complex data types, difficulty aligning multimodal data; challenges with processing 3D structures.
2. Limitations of machine learning: Requires extensive domain knowledge, performance limited by feature quality, poor generalization ability, strong dependency on data, unsuitable for complex data structures, struggles with increasingly rich multimodal data.
3. Limitations of deep learning: Demands large volumes of data, poor interpretability, requires powerful hardware for computational support, model tuning and optimization typically time-intensive.
4. Data Security and privacy concerns: The increasing richness of data sources raises concerns about the legitimate and fair use of personal data. Establishing legal and reliable data exchange centers, anonymizing personal data, and standardizing data resource management are critical challenges.

However, with the continuous advancement of AI methods and comprehensive hardware support, numerous cutting-edge approaches are emerging. These include using generative AI, quantum chemistry, and large models.

In the future, quantum chemistry methods can leverage particle-level information to handle molecular interaction data, optimize molecular structure design, and predict molecular reactions. Generative AI can learn from extensive data inputs to predict complex structures like protein folding, similar to AlphaFold, generating new compounds disruptively. Finally, large models applied in bioinformatics extend beyond simple question and answer systems, utilizing their inference capabilities for tasks such as molecular sequence analysis, structure prediction, and molecular interaction forecasting in an end-to-end multitask feedback system.

By integrating atomic information from quantum chemistry into complex computational systems of large models with sufficiently diverse sample data and corpora, breakthroughs at the particle level may enable profound developments, potentially leading us deeper into the microscopic world, beyond pharmaceutical discovery alone.

## Data Availability

Our article belongs to the survey category. The relevant statistical data in the article comes from references. We also cite them in the article.
